# Microvascular Dysfunction in Heart Failure With Preserved Ejection Fraction

**DOI:** 10.3389/fphys.2019.01347

**Published:** 2019-11-05

**Authors:** Domenico D’Amario, Stefano Migliaro, Josip A. Borovac, Attilio Restivo, Rocco Vergallo, Mattia Galli, Antonio Maria Leone, Rocco A. Montone, Giampaolo Niccoli, Nadia Aspromonte, Filippo Crea

**Affiliations:** ^1^Department of Cardiovascular Medicine, Fondazione Policlinico Universitario Agostino Gemelli IRCCS, Rome, Italy; ^2^Department of Pathophysiology, University of Split School of Medicine, Split, Croatia

**Keywords:** heart failure, precision medicine, preserved ejection fraction, microvascular dysfunction, diastolic abnormalitiy

## Abstract

Heart failure with preserved ejection fraction (HFpEF) is an increasingly studied entity accounting for 50% of all diagnosed heart failure and that has claimed its own dignity being markedly different from heart failure with reduced EF in terms of etiology and natural history (Graziani et al., 2018). Recently, a growing body of evidence points the finger toward microvascular dysfunction as the major determinant of the pathological cascade that justifies clinical manifestations (Crea et al., 2017). The high burden of comorbidities such as metabolic syndrome, hypertension, atrial fibrillation, chronic kidney disease, obstructive sleep apnea, and similar, could lead to a systemic inflammatory state that impacts the physiology of the endothelium and the perivascular environment, engaging complex molecular pathways that ultimately converge to myocardial fibrosis, stiffening, and dysfunction (Paulus and Tschope, 2013). These changes could even self-perpetrate with a positive feedback where hypoxia and locally released inflammatory cytokines trigger interstitial fibrosis and hypertrophy (Ohanyan et al., 2018). Identifying microvascular dysfunction both as the cause and the maintenance mechanism of this condition has opened the field to explore specific pharmacological targets like nitric oxide (NO) pathway, sarcomeric titin, transforming growth factor beta (TGF-β) pathway, immunomodulators or adenosine receptors, trying to tackle the endothelial impairment that lies in the background of this syndrome (Graziani et al., 2018;Lam et al., 2018). Yet, many questions remain, and the new data collected still lack a translation to improved treatment strategies. To further elaborate on this tangled and exponentially growing topic, we will review the evidence favoring a microvasculature-driven etiology of this condition, its clinical correlations, the proposed diagnostic workup, and the available/hypothesized therapeutic options to address microvascular dysfunction in the failing heart.

## Introduction

The burden of heart failure nowadays is constantly increasing and even if the most presentation is still characterized by the reduction in ejection fraction, the phenotype of heart failure with Preserved Ejection Fraction (HFpEF) is becoming constantly more common, mainly because of the improvement in diagnostic tools, the higher clinical awareness, and the steady increase in life expectancy. Accordingly, HFpEF now accounts for about 2.5 million cases in the United States, which means almost half of all the recorded heart failure diagnosis. Although the prevalence of this condition is growing worldwide, its complex pathophysiology is yet to be fully elucidated. For many years, it was simply considered a mere problem of myocardial stiffness, mostly due to chronic hypertension and increased circulatory demand, with elevated left ventricular filling pressures eventually resulting in concentric hypertrophy and consequent diastolic dysfunction (Lam et al., 2018). Nonetheless, this paradigm has recently been shifted, as researchers are disclosing several clues about the role of microvessels in the development of this syndrome, aiming to provide clinicians new tools to improve patient outcomes. Hereafter, we will review the major evidence supporting this hypothesis and the new therapeutic avenues arising from these new physio-pathological concepts.

### Epidemiology

HFpEF nowadays accounts for approximately half of the heart failure (HF) cases with incidence trends showing that it might become the most prevalent form of HF. In the general population, incidence of HFpEF shows a marked imbalance in sex prevalence (Beale et al., 2018), with elder women being more at risk than men (Dunlay et al., 2017). Generally, HFpEF phenotype prevalence increases with aging, particularly among patients older than 64 years, and in the third age might exceed the prevalence of HF with reduced ejection fraction (HFrEF) (Gomez-Soto et al., 2011; Savarese and Lund, 2017). Hypertension, obesity, coronary artery disease, diabetes mellitus, atrial fibrillation (AF), obstructive sleep apnea (OSA), and dyslipidemia are highly prevalent phenomena in HFpEF, underscoring the causal role of comorbidities in the pathophysiology of this phenotype. In particular, arterial hypertension was long thought to be the major driver in HFpEF development (Bhuiyan and Maurer, 2011). Clinical outcomes in HFpEF are poor, marked by the high burden of hospitalizations and mortality rates, mostly related to non-cardiovascular causes (Ponikowski et al., 2016), confirming once more the pivotal role of comorbidities in this syndrome.

### Diagnosis

Within the considerably wide spectrum of HF presentations, HFpEF undoubtedly covers the slice where the diagnosis is most challenging. Recognizing HFpEF may be complex since clinical manifestations are not specific, requiring a high degree of clinical suspicion. Indeed, the strong multimorbidity background in HFpEF often imposes diagnostic dilemmas because HF-like symptoms might be attributed to non-cardiac concomitant causes and diseases. Moreover, primary HFpEF must be distinguished from diastolic dysfunction not directly arising from myocardial disease but secondary to bystander cardiac conditions (e.g., valvular disease, pericardial disease, etc.) (Del Buono et al., 2018).

It has lately been recognized that patients with obesity, decreased exercise capacity, increased cardiac filling pressures, and normal left ventricular ejection fraction (LVEF) do not suffer from a homogeneous disorder but might present with at least three phenotypes of heart failure that should be clinically differentiated since they might have different response to available therapeutic armamentarium (Packer, 2019).

According to 2016 ESC guidelines on HF diagnosis and treatment, diagnosis can be made in the presence of symptoms and/or signs of heart failure, a LVEF ≥50%, increased levels of natriuretic peptides and either the presence of structural heart disease and/or diastolic dysfunction.

According to the ESC guidelines, the two recommended biomarkers are N-terminal pro B-type natriuretic peptide (NT-proBNP cut-off value for diagnosis >125 pg/ml) and brain natriuretic peptide (BNP, cut-off >35 pg/ml) (Ponikowski et al., 2016).

NT-proBNP strongly pairs the increase in LV diastolic filling pressure (Tschope et al., 2005), has a very high negative predictive value, and both baseline value and changes over time have relevant prognostic implications. In the chronic setting of HfpEF, natriuretic peptides are usually lower than HFrEF and may even drop to normal or near normal values during the symptom-free periods; this is consistent with the smaller amount of wall stress, according to Laplace law, in the presence of a small and thick ventricle, in opposition with the dilated and thin chamber in HFrEF.

Nonetheless, none of the cut-off values considered can discriminate between the two types of HF (HFrEF vs. HFpEF). Recently, galectin-3 and soluble ST-2 are showing promising results mostly in terms of outcome prediction in HFpEF with the first one having received a class IIB recommendation for risk stratification in 2013 ACC/AHA guidelines. Additionally, several circulating molecules (TNF-a, IL-6, cystatin C, GDF-15, TIMPs, etc.) are currently being investigated in order to evaluate the possible clinical and prognostic implication. So far, contradictory results have been produced possibly due to the different contexts and the heterogeneous HFpEF pathophysiology (Meijers et al., 2016).

In HFpEF, indeed, the main structural alterations include left atrial volume index (LAVI) >34 ml/m^2^ or a left ventricular mass index (LVMI) ≥115 g/m^2^ for males and ≥ 95 g/m^2^ for females. Functional echocardiographic abnormalities are mostly focused on the signs of diastolic dysfunction such as the ratio between early mitral inflow velocity and mitral annular early diastolic velocity (E/e′) being equal or higher than 13 and/or mean septal and lateral wall mitral annular early diastolic velocity (e′) being <9 cm/s. Other indirect functional abnormalities that can aid in the echocardiographic diagnosis of HFpEF are abnormalities in the global longitudinal strain (GLS) or elevated tricuspid regurgitation velocities (TRV), whereas speckle tracking echocardiography has been shown to provide early signs of LV diastolic dysfunction in a large animal model of metabolic dysfunction (van den Dorpel et al., 2017). In the case of discordant or inconclusive evidence coming from the imaging assessment of HFpEF patients, an echo stress test or an invasive assessment of LV filling pressures might be required (Ponikowski et al., 2016). Moreover, despite cardiac catheterization being largely essential in most of the complex cases, the diagnostic issue could be facilitated by using cardiac magnetic resonance imaging (cMRI) as T_1_ mapping cMRI is able to reliably measure diffused interstitial fibrosis that robustly correlates with an invasively measured LV stiffness (Rommel et al., 2016).

### Pathophysiology

Since the original description of HFpEF, the relation between the clinical syndrome and the presence of comorbities has been described: epidemiological studies and prospective clinical trials showed consistently that hypertension, especially in the elderly, and irrespective of the administration of blood pressure medications (Redfield et al., 2003; Zanchetti et al., 2007) was associated with echocardiographic signs of diastolic dysfunction.

Certainly, the high workload weighting on the heart of hypertensive patients triggers a structural remodeling which can be an important determinant of diastolic failure, but the long-lived “mostly mechanic” paradigm of increased afterload as the major, if not the only, mechanism underlying the pathophysiology of this condition has been recently challenged. In recent studies, hypertension-related diastolic dysfunction was already contextualized in a more “systemic” view, with the evidence of paracrine and autocrine signaling of the renin-angiotensin-aldosterone system (RAAS), involved both in inappropriate blood pressure elevation and reactive ventricular hypertrophy, myocardial fibrosis, vascular inflammation, and dysfunction (Sun et al., 2004; Sciarretta et al., 2009). In 2013, Paulus and colleagues proposed a more multifaceted model (Paulus and Tschope, 2013), in which comorbidity-associated systemic inflammation is the main driving factor impairing myocardial performance by inducing, as a “primum movens,” the microvascular dysfunction. This approach is at variance with the one traditionally proposed for patient with HFrEF.

This new paradigm, having the microvascular dysfunction at the center of the disease evolution, has gained support over years. Recently, Graziani and Crea proposed an innovative theory that identifies microvascular dysfunction as the “common soil” for the occurrence of both microvascular angina and HFpEF (Graziani et al., 2018) (see below).

The logical consequence is that, having dissected different mechanisms in HFpEF and HFrEF, the therapeutic strategies implemented might differ due to the pivotal role of microvascular dysfunction in HFpEF, thus this might act as a treatment target (Gong et al., 2018).

Irrespective of the primer of the syndrome, its hemodynamic consequences are well-defined. The first step in the progression of HFpEF into a clinically significant disease is the alteration in diastolic function marked by both impairment of active relaxation process and an increase in the ventricular stiffness during the passive filling phase (Zile et al., 2004). In the absence of mitral valve disease, these changes directly cause a rise in left atrial pressure, leading to adverse remodeling of this chamber. The most obvious hemodynamic consequence of chronically elevated LV filling pressure is the parallel increase in pulmonary artery systolic pressure (PASP) which has been reported in 40–80% of patients with HFpEF and it is associated with more symptoms and a poorer outcomes (Lam et al., 2009). The increase in pulmonary artery diastolic pressure is also associated with a transition toward acute decompensation of HF (Zile et al., 2008), eventually resulting in right heart dysfunction, leading to biventricular HF. The combined loss of pulmonary and cardiac reserve, together with the reduced peripheral oxygen delivery and extraction is the cause of reduced exercise tolerance, which is a major feature of HF (Del Buono et al., 2019).

### Inflammation, Microvascular Dysfunction, and Heart Failure With Preserved Ejection Fraction

Even if the hemodynamic and clinical features of HFpEF are reasonably well-described, the complex molecular and physiological mechanisms underlying HFpEF are still poorly understood. The paradigm shift from the traditional *“overload model”* to the emerging *“microvascular hypothesis”* was initially hinted by the observation that patients from ALLHAT trial who developed HFpEF were better identified by the elevated body mass index (BMI) rather than elevated arterial pressure, suggesting that comorbidities could somehow blunt heart function in a more complex way than previously thought (Davis et al., 2008). Nowadays, the most credited hypothesis proposes that the high burden of comorbidities triggering a systemic pro-inflammatory state impairs the physiology of the endothelium and subsequently the perivascular environment activates complex molecular pathways that eventually converge to myocardial fibrosis, resulting in a significant stiffening of the ventricle and diastolic dysfunction (Paulus and Tschope, 2013; Ohanyan et al., 2018), as suggested by the elevation of circulating inflammatory biomarkers (IL-1RL1, CRP, GDF15, TNF-α, sST-2, pentraxin-3, etc.) (Cheng et al., 2013; D’Elia et al., 2015; Sanders-van Wijk et al., 2015), interestingly even in a greater magnitude than observed among patients with HFrEF (Matsubara et al., 2011; Shah et al., 2011; Sanders-van Wijk et al., 2015). Further clue in this regard is the observation of elevated expression of inflammatory endothelial adhesion molecules in LV endomyocardial biopsies from patients with HFpEF, together with the high rate of inflammatory-related morphological changes affecting other organs (lungs, kidneys, and skeletal muscles) (Lam et al., 2018). Furthermore, high-sensitivity C-reactive protein (hs-CRP) levels in HFpEF patients are associated with the greater disease burden, thus supporting the idea of comorbidity-driven inflammation (DuBrock et al., 2018).

As demonstrated in experimental models of diabetes, obesity, and metabolic cardiomyopathies, a systemic inflammation affects local cardiac endothelial function by impairing nitric oxide (NO) pathway comprising cyclic guanosine monophosphate (cGMP) and activated protein kinase G (PKG) (Paulus and Tschope, 2013). Diabetic cardiomyopathy could be used as a paradigm to outline this complex picture in which microvascular driven-diastolic dysfunction results from the conjunction of the production of advanced glycation end-products (AGEs) that modify the composition of extracellular matrix (ECM) by activating profibrotic transforming growth factor beta (TGF-β) pathway. At the same time, increased oxidative stress, secondary to reactive oxygen species (ROS) production and lipotoxicity, disrupts nitric oxide (NO) metabolism, while titin molecule changes its isoform phenotype ultimately leading to myocardial stiffness through the activation of PI3K/AKT downstream target of abnormal insulin receptor signaling (Meagher et al., 2018).

This proinflammatory milieu implies elevated levels of IL-6, TNF-α, sST-2, pentraxin-3 and changes in endothelial adhesion molecular phenotype with the increased expression of vascular cell adhesion protein 1 (VCAM-1) and E-selectin which together promote monocyte migration (Westermann et al., 2011). Eventually, the consequent local ROS accumulation transforms NO into peroxynitrite cutting down its bioavailability (Schulz et al., 2008). As a consequence, downstream soluble guanylate cyclase (sGC) function, and thus cGMP-induced protein kinase (PKG) activation, is reduced, causing an increase in the cardiomyocyte resting tension secondary to hypophosphorylation of titin and abolition of the antihypertrophic and antifibrotic effects of PKG.

Indeed, PKG acts as an endogenous physiological brake on the negative remodeling effects mediated by TGF-β by phosphorylating SMAD complexes (Li et al., 2007; Westermann et al., 2011; Paulus and Tschope, 2013). Interesting studies on swine model of hypertension, diabetes, obesity, and hypercholesterolemia by Sorop et al. recently confirmed that comorbidities can rapidly prime this complex process heading to microvascular dysfunction and, consequently, myocardial diastolic dysfunction (Sorop et al., 2018). Furthermore, these studies highlighted a modulation of the vasodilators/vasoconstrictors imbalance throughout progression of coronary microvascular dysfunction (CMD), thus suggesting a time dependence of the molecular phenotype. Particularly, the NO impairment has been noted early in the disease, whereas progression implies a prevailing role by endothelin-1-mediated vasoconstriction (van den Heuvel et al., 2011; Sorop et al., 2016, 2017). To further emphasize the importance of endothelium and microvessels in HFpEF, a recent link between *Parvovirus B19* infection of these cells and impaired diastolic function with poor response to acetylcholine tests has been observed (Tschope et al., 2005).

**Figure 1:**
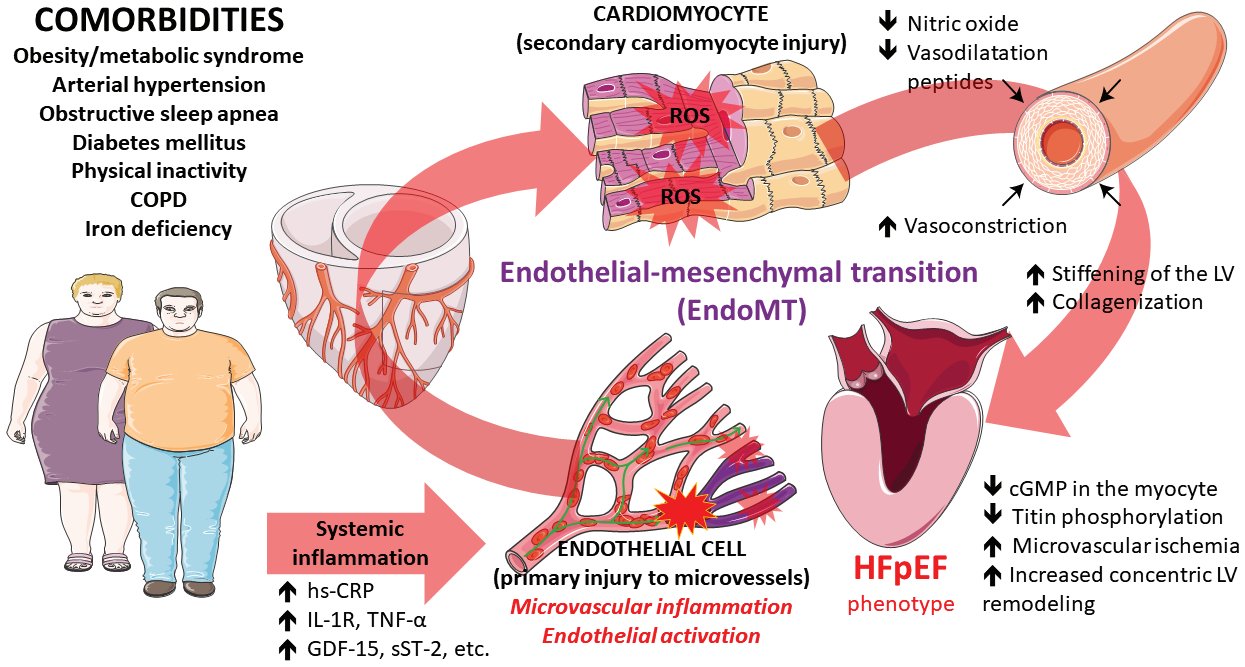
Pathophysiological pathways that contribute to the development of heart failure with preserved ejection fraction converging at the level of perivascular and microvascular inflammation and endothelial activation ultimately leading to myocardial dysfunction. Some illustration elements used in this figure have been kindly provided by the Servier. Servier Medical Art is licensed under a Creative Commons Attribution V.3.0 Unported License.

Of note, circulating levels of irisin, a recently discovered myokine inversely correlated with the total antioxidant capacity among patients with HFpEF while this effect was not observed in patients with HFrEF further suggesting two distinct pathophysiological mechanisms involved in these HF phenotypes (Silvestrini et al., 2019). This study also established a hypothesis that the level of oxidative stress modulates irisin secretion in HFpEF. Finally, network analysis showed that biomarker profiles specific for HFpEF are related to inflammation and extracellular matrix reorganization, as opposed to biomarker profiles related to cellular proliferation and metabolism as determined in HFrEF, further making a distinction between these two HF entities (Tromp et al., 2018). The proposed scheme focused on inflammation, microvascular dysfunction, and onset/progression of HFpEF is depicted and summarized in Figure 1.

#### Endothelium and Microvessels

Coronary microvascular dysfunction (CMD) comprises structural and functional abnormalities affecting heart microcirculation in different clinical conditions, not only as a mere epiphenomenon, but also as a pathogenetic factor and prognostic marker. The first acknowledgment of microvascular role in heart disease came from the observation that almost 50% of patients with stable angina and 10% of patients presenting with myocardial infarction do not have obstructive epicardial CAD: in both cases, CMD could be the hidden culprit causing symptoms. Camici and Crea have classified CMD on the basis of clinical context in which it occurs, obtaining four main types: (1) CMD in the absence of obstructive CAD and myocardial disease; (2) CMD in the presence of myocardial disease; (3) CMD in the presence of obstructive CAD; (4) iatrogenic CMD (Camici and Crea, 2007).

Nowadays, we are gaining more understanding that the burden of CMD is larger than previously thought, going beyond ischemic heart disease and involving HFpEF patients. It is recognized that in this syndrome, there is an increase in stiffness and resting vasoconstriction of large vessels, mainly due to increased focal adhesion between vascular smooth muscles cells and to the slower kinetics of the non-muscle myosin isoform that could become overexpressed in these cells (Contard et al., 1993; Lyle and Brozovich, 2018). These pathological changes in great vessels reflect the presence of endothelial dysfunction. Moving from this hypothesis, different studies showed that diastolic impairment, the major functional abnormality in HFpEF, is related to coronary endothelial dysfunction, independently from the presence of CAD (Shantsila et al., 2012). Systemic microvascular impairment was indirectly evaluated in HFpEF by flow-mediated dilation (FMD) of brachial artery, showing altered endothelium-dependent responses; moreover, not only FMD was absolutely reduced but it was lower than those of hypertensive controls, classically considered at higher risk of HFpEF, thus reinforcing the idea that increased afterload is not sufficient *per se* to drive HFpEF evolution (Lee et al., 2016). To further lend support to the primary role of micro vessels in HFpEF, many studies assessed important differences in reactive hyperemia index (RHI), which more specifically identifies the presence of microvascular dysfunction and proving to have prognostic value in this population (Hundley et al., 2007; Haykowsky et al., 2013; Matsue et al., 2013; Lee et al., 2016). A previous study also demonstrated the link between reduced global cardiovascular reserve measured by reactive hyperemia index and exercise symptoms in HFpEF (Borlaug et al., 2010). These observations were recently supported by Dhakal and colleagues who reported a significant reduction in peripheral oxygen extraction during exercise in patients with HFpEF affirming the involvement of microvascular dysregulation in the HFpEF symptom onset (Dhakal et al., 2015).

Data on impaired microvascular function has been indirectly assessed also by invasive studies, showing reduced thrombolysis in myocardial infarction (TIMI) frame count and myocardial blush grade in HFpEF patients without overt CAD (Sucato et al., 2015). Furthermore, functional studies performed with the us pressure wire, demonstrated a linear correlation between the index of microvascular resistance (IMR) and LV end-diastolic pressure (LVEDP) (Dryer et al., 2018) and a reduced endothelium-dependent vasodilator response to acetylcholine in HFpEF patients (Balmain et al., 2007). Finally, Taqueti et al. documented that reduction in coronary flow reserve (CFR) closely related to HFpEF diagnosis and, together with diastolic dysfunction significantly predicted the risk of major adverse cardiovascular events (MACE) and hospitalizations due to HF (Taqueti et al., 2018). Interestingly, CFR impairment was also related to the presence of detectable troponin levels (Taqueti et al., 2018). The significant decrease of CFR in HFpEF was further confirmed by non-invasive imaging modalities such as contrast cine-MRI (Kato et al., 2016), and more recently by the PROMIS-HFpEF study that was able to validate the correlation between Echo-CFR and reactive hyperemia index (RHI), revealing a high prevalence of CMD in HFpEF patients (Shah et al., 2018). Besides these functional abnormalities, it has been shown that microcirculatory derangement also comes from structural changes in coronary microvascular density (MVD) (Hoenig et al., 2008). Indeed, post-mortem study by Mohammed and colleagues showed a significant decrease in MVD in HFpEF patients, regardless of the presence of epicardial CAD; furthermore, lower MVD correlated to greater myocardial fibrosis (Mohammed et al., 2015).

Therefore, physiology behind the increase in IMR and reduction in CFR probably rests on both the rarefaction in capillary concentration and the reduced bioavailability of NO (Xu et al., 2018).

The obvious consequence of microvascular dysregulation is the presence of areas of local chronic ischemia. Accordingly, several studies proposed latent ischemia as the center of a vicious circle in which reduction in oxygen delivery impairs myocyte relaxation, whereas diastolic dysfunction promotes intramyocardial tension, increasing oxygen consumption demands (Pepine et al., 2014; Ikonomidis et al., 2015; Tschope and Post, 2015; Crea et al., 2017). Additionally, coronary microvascular ischemia further supports inflammation through release of ROS, thus closing the vicious cycle. The microvascular hypothesis of HFpEF finds more support if we transcend the anatomic boundaries of the heart: while it is known that chronic kidney disease (CKD) strictly pairs with HFpEF by fostering inflammation through uremic toxins increase, urinary sodium retention, and altered levels of endocrine factors (Ter Maaten et al., 2016), Shah and colleagues described a link between CMD and CKD through the evidence that patients with worse CFR have a higher urinary albumin-to-creatinine ratio (ACR) (Shah et al., 2018).

#### Titin

Titin is an elastic protein responsible for LV passive stiffness, as it connects the Z-disk of sarcomere to the M-band, thus preventing overstretching. Diastolic left ventricular (LV) stiffness in HFpEF occurs primarily due to the changes in sarcomeric titin. In this context, an isoform shift, from the more compliant isoform *N2BA* to the stiffer isoform *N2B* (Kruger and Linke, 2009), has been observed, and together with titin hypophosphorylation attributable to PKG inactivity (Kruger et al., 2009), leads to reduced elastance (compliance) of myocytes (van Heerebeek et al., 2012).

#### Calcium Overload

Diastolic dysfunction in HFpEF is characterized not only by the increased LV passive stiffness but also by impaired myocardial active relaxation resulting from intracellular calcium overload: in the setting of HF, there is an increase in late sodium currents leading to elevated intracellular Na^+/^Ca^2+^ exchange activity resulting in excessive cytosolic calcium load during the phase of diastole (Senni et al., 2014). The mechanism of calcium overload with the subsequent increase in oxidative stress may be facilitated by the upregulation of RAAS system and appears to be strictly related with fibrotic remodeling (Shahbaz et al., 2010).

#### Cellular Senescence

Senescence is a state of cell-cycle arrest normally related to aging and induced by mitogenic signals, DNA damage or inflammation. Recently, cellular senescence was described as a possible factor involved in microvascular dysfunction by triggering the vicious cycle of inflammation and senescence driving HFpEF pathophysiology. Animal model engineered to simulate a senescent cellular phenotype had reduced NO-dependent endothelial signaling, both because of reduced basal NO levels and reduced eNOS function; moreover, these animals had increased levels of fibrosis driven by local inflammation (Gevaert et al., 2017).

#### Extracellular Matrix

Moving to the non-cellular compartment, the extracellular matrix in HFpEF represents an imbalance between collagen deposition and degradation, resulting in interstitial fibrosis, promoted by endothelial-mesenchymal transition (EndoMT), induced by TGF-β (Ter Maaten et al., 2016) pathway activation. EndoMT is becoming increasingly recognized as the driving pathogenetic force behind fibrotic disorders marked by the loss of specific markers by endothelial cells thereby acquiring mesenchymal or myofibroblastic phenotype ultimately leading to increased expression of cell products such as α smooth muscle actin (α-SMA) and type I collagen (Piera-Velazquez et al., 2011, 2016; Cho et al., 2018). Furthermore, Sniegon and colleagues demonstrated that EndoMT in hypoxic microvascular endothelial cells might induce *via* paracrine mechanisms cardiomyocyte apoptosis through TGF-β_1_ and SMAD signaling (Sniegon et al., 2017). EndoMT has been hypothesized to generate a substantial number of cardiac fibroblasts in response to pressure overload-induced myocardial injury thus being labeled as a promising therapeutic target (Moore-Morris et al., 2014) and the key link in the interaction between inflammation and endothelial dysfunction (Cho et al., 2018): when EndoMT is developing, there is not only a quantitative increase in total collagen content, but also a disproportion between its various isoforms, with an overexpression of collagen type I relative to type III, and an increased degree of crosslinking between collagen molecules (Querejeta et al., 2004; van Heerebeek et al., 2006). Interestingly, the myocyte and the extracellular compartment seem to be interconnected equally contributing to diastolic stiffness (Kakkar and Lee, 2010). It has also been speculated that repeated episodes of subclinical micro-ischemia cause focal areas of fibrosis finally promoting HFpEF. This phenomenon could explain the evidence of a low coronary flow reserve as predictor of diastolic dysfunction in patients with microvascular angina (Taqueti et al., 2018).

#### Adipose Tissue

An important role in inflammation and microvascular dysfunction may be related to the accumulation and inflammation of epicardial adipose tissue that ultimately leads to fibrosis (Obokata et al., 2017). In chronic inflammatory conditions such as HFpEF, epicardium has become the source of deranged adipogenesis causing increased production of proinflammatory adipokines that can cause both atrial and ventricular fibrosis thus proposing the role of epicardial adipose tissue as the transducing component that mediates adverse systemic and metabolic effects onto the heart (Packer, 2018c). Lastly, plasma volume expansion especially seen in case of high BMI patients has been proposed as a cause of persistent cardiac volume overload (Maurer et al., 2005).

#### miRNA

The latest frontier in the pathophysiology of HFpEF involves the role of miRNA in inflammation and endothelial dysfunction: miRNA 146 and 155 seem to be relevant for the inflammatory cytokine production and the nuclear factor kappa-light-chain-enhancer of activated B cells (NF-kB) activation. miRNA 126 is instead implied in the loss of endothelial homeostasis and vascular integrity, that leads to “leaky vessels,” correlated to VCAM expression and increased monocyte recruitment (Rech et al., 2018).

#### Insulin Signaling in Heart Failure

It has been proposed that increased insulin signaling might exert adverse effects on cardiac remodeling, vasculature, kidneys, and adipose tissue, thus predisposing an individual with diabetes to HF and HFpEF in particular (Packer, 2018d). Clinical trials showed that drugs that potentiated insulin signaling such as sulfonylureas, thiazolidinediones, and incretins were associated with an augmented risk of HF development or worsening of the preexisting HF, while antidiabetic drugs that counter hyperglycemia through non-insulin pathways such as metformin and sodium-glucose cotransporter 2 inhibitors (SGLT-2 inhibitors) decreased the risk of HF development (Nassif and Kosiborod, 2018; Packer, 2018d). On the other hand, effects of glucagon-like peptide-1 (GLP-1) signaling are responsible for stimulation of insulin release in the pancreatic β-cells, thus alleviating hyperglycemia, while the inhibition of neprilysin with agents such as sacubitril is implicated in the augmentation of GLP-1 signaling which might have a clinical relevance in patients with HF, both with or without diabetes (Packer, 2018b).

#### Obstructive Sleep Apnea and Heart Failure With Preserved Ejection Fraction

Obstructive sleep apnea (OSA) is known to be marked by sleep disturbance, chronic intermittent hypoxia, hemodynamic changes, and sympathetic activation and this might contribute to development or sustaining of the HF (Cowie, 2017). According to latest data, prevalence of obstructive sleep apnea (OSA) in HFpEF was 16.8%, and patients with OSA were more likely to develop HFpEF compared to those without OSA (Abdullah et al., 2018). Furthermore, OSA was independently associated with an increased risk of admission with HFpEF (relative risk = 2.2, 95% CI: 2.12–2.21) (Abdullah et al., 2018). Furthermore, treatment of sleep-disordered breathing in HFpEF was associated with significant reductions in left atrial volume suggesting an improvement in diastolic function (Daubert et al., 2018). Finally, OSA, even if properly treated, might worsen long-term cardiac function and outcomes in patients with HFpEF with higher levels of brain natriuretic peptides being documented among HFpEF patients with OSA compared to those without (Arikawa et al., 2016).

#### Iron Metabolism and Heart Failure With Preserved Ejection Fraction

Iron is an important micronutrient that acts as a catalyst for many biochemical reactions and, as an integral component of hemoglobin plays a key role in tissue oxygenation as well as the oxygen-binding in skeletal muscles and myocytes (Mordi et al., 2018). Iron deficiency (ID) is a common comorbidity in HFpEF and is associated with reduced exercise capacity (as measured by 6-min walk test) and reduced quality of life (as measured by EQ 5D questionnaire) (Bekfani et al., 2019), thus is an established correlate of other adverse clinical outcomes (Saraon and Katz, 2016), negatively affecting VO_2max_ (Martens et al., 2018). Furthermore, patients with ID have more severe diastolic dysfunction than those without, with an incremental increase in the severity of diastolic dysfunction proportionate to the level of ID. It seems that absolute ID (both serum iron and ferritin decreased) rather than functional ID (low serum iron but serum ferritin within 100–299 μg/L and transferrin saturation < 20%) is associated with increased risks of one-year mortality or HF admissions in patients with HF (Nakano et al., 2018). In HFpEF patients without anemia, cardiac dysfunction and impaired exercise capacity were phenomena occurring independently of functional ID, as confirmed in the study of Kasner et al., 2013. Intravenous iron supplementation has been proven to improve functional capacity of HF patients, while anemia correction seemed to be similar between oral iron and intravenous iron, as demonstrated in IRON-HF study (Beck-da-Silva et al., 2013). Currently ongoing FAIR-HFpEF trial will provide further insights on the effects of intravenous iron (ferric carboxymaltose) administration among patients with HFpEF in regards to exercise tolerance, symptoms, and quality of life (NCT03074591) (Mordi et al., 2018).

### Therapeutic Potential of Coronary Microvascular Dysfunction in Heart Failure With Preserved Ejection Fraction

Many therapeutic approaches in HFpEF are rendered from the experience of HFrEF patients, but given the different pathophysiological mechanisms of these syndromes, the pharmacological strategies focused on the blockade of the noradrenergic and renin-angiotensin-aldosterone system (RAAS), have been consistently shown to be less effective in HFpEF. Cleland et al. suggested that beta-blockers conferred similar beneficial effects in patients with HFrEF and those with midrange LVEF (HFmrEF) phenotype, while these effects were absent in patients with preserved LVEF implicating that different targets might exist in patients with preserved vs. impaired systolic function (HFmrEF being a milder form of systolic dysfunction) (Cleland et al., 2018). With microvascular dysfunction having a more significant role in HFpEF than in HFrEF, drugs that could act on small vessels and microenvironment function could open new frontiers in alleviating HFpEF symptoms, functional limitations, and prognosis.

### Nitric Oxide /Cyclic Guanosine Monophosphate /Protein Kinase G Pathway

Given the breakdown in NO metabolism is likely to be the cause of endothelial dysfunction in HFpEF, it is reasonable to propose that organic NO donors could be a potentially useful therapeutic tools (Lacolley et al., 2001; Bauersachs et al., 2002). Unfortunately, results from the early studies of these drugs are at the best inconclusive.

Redfield and colleagues showed in the NEAT-HFpEF trial that isosorbide mononitrate (ISMN) did not improve but, rather, tended to reduce the total physical activity level with no improvement in submaximal exercise capacity. This evidence conflicts with the microvascular hypothesis of HFpEF, but it must be noticed that in this cohort of patient, there was a high incidence of hypotension, a common adverse reaction to this drug class, which could have limited effort tolerance in enrolled patients which are extremely sensitive to alterations in their hemodynamic state (Redfield et al., 2015).

On the other hand, the inorganic form of NO donor, nitrite, seems to have some benefits, improving arterial stiffness, diastolic LV pressures, and pulmonary artery pressure during exercise (Borlaug et al., 2015; Zamani et al., 2015). Trials of oral nitrite (ONOH) and nitrate (KNOCK-OUT HFpEF trial) are currently ongoing, although INDIE-HFpEF study failed to show an improvement in functional capacity with nebulized administration of inorganic nitrite (Patel and Shah, 2019). Moreover, some data suggest that nitroxyl donors may directly act on myofilaments, and subcellular SERCA2a and RyR2 components within the myocyte, leading to positive inotropic and lusitropic effects and increased diastolic cell length (Shah et al., 1994; Gao et al., 2012). However, caution is warranted, as some evidence points at inorganic nitrates as a culprit rather than a treatment of endothelial dysfunction by causing a local release of free oxygen radicals; furthermore, after their administration, increased expression of endothelin has been observed (Oelze et al., 2013).

Therefore, although inorganic NO donors seem like an attractive therapeutic option in tackling LV stiffness in HFpEF, major limitations in their use currently exist, such as tachyphylaxis, impaired bioactivation of nitrates group, and the important preload reduction secondary to the peripheral vasodilation exerted by these agents.

Given the apparently narrow *risk-reward* ratio of previously described drugs, research focused on direct stimulation of NO-activated guanylyl cyclase (GC) by the experimental drug BAY41-8543, with preliminary data on animal model observing the beneficial role in terms of morbidity and mortality in HFpEF; interestingly, these advantages were independent of hypertrophy reversal (Wilck et al., 2018). Such observations reinforced the role of the NO/PKG cascade impairment and microvascular dysfunction in HFpEF.

On this hypothesis rests, the SOCRATES-HFpEF trial, in which *vericiguat*, a once-daily stimulator of sGC, was tested in population of patients with preserved systolic function; the primary endpoint, defined as the change in NT-proBNP level, was negative, but there was an evidence of improved physical capacity and quality of life (Pieske et al., 2017). One of the speculations to justify this discrepancy is that sGC stimulators reach different tissue concentrations in various organs involved in HFpEF, acting on skeletal muscles more efficiently than on the heart; another option is that patients in the study were enrolled too close to a recent hospitalization and were likely to have an advanced disease stage (Patel and Shah, 2019).

The last actors in the NO metabolism chain are phosphodiesterases (PDEs), enzymes responsible for cyclic GMP (cGMP) degradation. Ideally, suppressing PDEs activity would thus increase cGMP levels independently from NO stimulation, thereby leading to improved microvascular regulation. Clinical hopes were high when it was documented that a PDE-5a inhibitor administered to an animal model of HFpEF improved LV relaxation and reduced diastolic LV stiffness (Bishu et al., 2011). However, further two major trials performed in humans that assessed the role of PDE-5 inhibitors in HFpEF failed to show real advantages on clinical outcomes, likely because of the detrimental effects of the steep and abrupt blood pressure lowering, as seen in this population, which moreover led to raised plasma creatinine and urea levels (Redfield et al., 2013; Hoendermis et al., 2015). Moreover, enrolled patients had a high prevalence of atrial fibrillation and elevated NT-proBNP levels, signs of already advanced disease (Kovacs et al., 2016). Nonetheless, sildenafil was reported as a beneficial agent in terms of right ventricular function and pulmonary pressure regulation in HFpEF patients with documented pulmonary hypertension. Thus, despite contradictory data, sildenafil still remains a viable option, but should probably only be used in a special patient subpopulation with the specific profile associated with relevant comorbidities.

The fortune of PDE inhibitors resurged when PDE-9 inhibition proved to be superior to the inhibition of other, more widely known isoform. Indeed, upregulation of this protein was seen in LV biopsies in HFpEF patients and its blockage in HFpEF animal models regressed hypertrophy, fibrosis, and vascular dysfunction. Likely, the key of the superiority of PDE-9 inhibition compared to PDE-5 inhibition could be in the ability to modulate cGMP signaling independently from NO pathway, whereas PDE-5 inhibition still requires active synthesis of NO (Lee et al., 2015). It has been even postulated that a reactive overexpression of PDE-9 after administration of PDE-5 inhibition may neutralize the effect of PDE-5 pharmacological blockade. Further studies are required in this regard.

### Statins

Statins have gained the attention of researchers for both their well-known anti-inflammatory action and their pleiotropic effects on endothelial homeostasis that went beyond established cholesterol-lowering effects. Endothelial effects of statins mainly consisted of restoring NO downstream signaling and improving endothelium-dependent relaxation through the amelioration of redox balance. The molecular benefit of statin-treated HFpEF patients has been highlighted by endomyocardial biopsy data showing less myocardial nitrotyrosine presence, higher myocardial PKG activity, less cardiomyocyte hypertrophy, and lower cardiomyocyte resting tension (Paulus and Tschope, 2013). Recent data acquired from meta-analysis and observational studies agree that statins may reduce mortality in HFpEF patients (Fukuta et al., 2016; Tsujimoto and Kajio, 2018; Marume et al., 2019), particularly in the absence of CAD (Tsujimoto and Kajio, 2018; Marume et al., 2019). In concordance with the systemic background of HFpEF proinflammatory state, the statin-treated group showed a significantly lower incidence of non-cardiac death and HF hospitalization events, even without effect on cardiac death. The beneficial effect of statins on mortality did not have any significant interaction with cholesterol level or HF severity (Marume et al., 2019). Moreover, it has been observed that statins act to reduce the quantity of epicardial adipose tissue and decrease its proinflammatory activity by reducing the deposition of oxidized lipoproteins along with microcirculatory dysfunction (Paraskevaidis et al., 2012; Yamada et al., 2017; Packer, 2018a). Notwithstanding the need for confirmation from large RCTs, latest studies definitely suggest that statins should be considered for introduction in the clinical practice in this setting.

### Ranolazine

Ranolazine inhibits the increased late Na^+^ current and therefore may minimize intramyocyte Na^+^ accumulation and the resultant Ca^2+^ overload in HFpEF (Senni et al., 2014). This drug reduced diastolic tension in failing human heart and improved diastolic function in non-infarcted ischemic myocardium, isolated myocardium from failing human hearts and in chronic stable angina (Hayashida et al., 1994; Sossalla et al., 2008; Figueredo et al., 2011). It is, therefore, hypothesized that ranolazine may have beneficial effects in HF-PEF setting. The ranolazine for the treatment of diastolic heart failure (RALI-DHF) study is a proof-of-concept trial that evaluated the effect of ranolazine vs. placebo on general hemodynamics, indices of diastolic dysfunction, and biomarkers, in patients with HFpEF. After 30 min of infusion, significant decreases in LV end-diastolic pressure (LVEDP) and pulmonary capillary wedge pressure (PCWP) were observed, although invasively determined relaxation parameters and the non-invasive *E/e′* ratio were unaltered. These data justify additional studies of this drug in HFpEF (Jacobshagen et al., 2011).

### Anti-fibrotic Treatments

As proposed by Graziani and colleagues, HFpEF and idiopathic pulmonary fibrosis (IPF) share many similarities with microvascular dysfunction being a fundamental part of both disease evolution (Graziani et al., 2018). Therefore, many treatments approved in IPF could be useful in HFpEF, too. Nintedanib, one of the two drugs specifically approved for IPF, is an intracellular inhibitor of tyrosine kinase receptors, targeting platelet-derived growth factor (PDGF), fibroblast growth factor (FGF) and vascular endothelial growth factor (VEGF) receptor and other non-receptor tyrosine kinases (RTKs) of the SRC family that elicits antifibrotic responses on cardiac muscle in animal model of pulmonary disease (Rol et al., 2019). Although more specific studies are required to evaluate the safety and efficacy of this drug in HFpEF, it is a viable treatment target which warrants further investigations. Similarly, pirfenidone is a pyridine-like molecule with an unknown pharmacological target, expressing anti-fibrotic, anti-inflammatory, and antioxidant properties observed in animal studies (Lopez-de la Mora et al., 2015). Pirfenidone also showed direct inhibition of TGF-β signaling pathway mediated by the inhibition of the FGF pathway (Lopez-de la Mora et al., 2015; Lehtonen et al., 2016). This drug was tested only in animal models where it appeared to reduce ventricular remodeling (Su et al., 2014). While no results are yet available in humans, one study in HFpEF patients is currently ongoing (NCT02932566). Phase III trials of pirfenidone in IPF documented its acceptable safety profile, thus making it a suitable treatment option if its beneficial properties are confirmed in the setting of HFpEF (Costabel et al., 2017).

### Physical Exercise

Physical activity can mitigate the effects of excessive oxidative stress and reduce the level of total ROS produced, by slowing NADPH oxidase activity (Heinonen et al., 2015). It has been observed that HFpEF patients, to a large degree, present with moderate-to-severe exercise intolerance (Packer, 2019). Moreover, exercise and physical activity are proven to increase the amount of nitric oxide produced by NOS (Grijalva et al., 2008). Physical exercise has both therapeutic and preventive effects on lifestyle factors that impose pleiotropic effects on the pathophysiological mechanisms that define HFpEF (Pandey et al., 2018). It can also act as a gene modulator upregulating transcription of nuclear factor E2 which can mediate the expression of antioxidant response elements (Nordin et al., 2014). Finally, physical exercise has been shown to increase nitric oxide production and tissue blood perfusion in diabetic CM models (Cohen et al., 2008). A study by Boyes and colleagues showed that heavy-intensity priming exercise conferred favorable effects on pulmonary oxygen uptake kinetics and muscle oxygenation in patients with HFpEF further implicating that microvascular muscle oxygen delivery may limit exercise tolerance in HFpEF population (Boyes et al., 2019).

### Anti-IL-1 Targeting

IL-1 is a cytokine involved in local and systemic inflammatory processes which have a causal role in impairing both systolic and diastolic function in animal models (Van Tassell et al., 2013). Consequently, research interest has been focused on the advantages of blocking this inflammation-sustaining cellular pathway. Anakinra is an IL-1 blocker approved for the treatment of chronic systemic inflammatory disease. In patients with HFpEF and rheumatoid arthritis, inhibition of IL-1 receptor restores diastolic function early after treatment with this agent (Van Tassell et al., 2013; Ikonomidis et al., 2015). Recently in the D-HART pilot study, in patients with HFpEF and high levels of CRP, anakinra led to a significant reduction in systemic inflammation and a measurable gain in exercise capacity (Van Tassell et al., 2014). The reduction in CRP levels correlated with the improvement in peak VO_2_, again certifying the role of inflammation in clinical manifestations of heart failure (Van Tassell et al., 2013).

### Mineralocorticoid Receptor Antagonists and Angiotensin Receptor Blockers

Chronic activation of RAAS exerts an important role in initiation and maintenance of HF leading to increased aldosterone levels and consequent collagen accumulation and endothelial dysfunction (Edelmann et al., 2013; Nair and Deswal, 2018). It is known that spironolactone can improve fibrosis by reducing extracellular matrix turnover and enhance endothelial vasomotor dysfunction (Lacolley et al., 2001). But so far, evidence on the efficacy of treatments targeting aldosterone is inconclusive.

In Aldo-DHF trial on spironolactone, it was shown that the improvement in diastolic function was associated with reverse LV remodeling; nevertheless, no effect on maximal exercise tolerance and patient symptoms were observed (Edelmann et al., 2013). The TOPCAT trial was designed to test the efficacy of spironolactone in reducing the incidence of the primary composite outcome of death from cardiovascular causes, aborted cardiac arrest, and hospitalization for HF. After a mean follow-up of 3.3 years, there was 5.9 events per 100 person-years in the spironolactone group and 6.6 events per 100 person-years in the placebo group (hazard ratio, 0.89; 95% confidence interval, 0.77–1.04; *p* = 0.14). Although a nominally significant reduction in the secondary outcome of hospitalization for heart failure was observed with spironolactone, overall the results of the TOPCAT trial were neutral.

It should be acknowledged that when the authors analyzed the data according to the eligibility stratum (natriuretic peptide stratum vs. hospitalization stratum), treatment with spironolactone was beneficial in patients who were enrolled in the natriuretic peptide stratum but not those in the hospitalization stratum (Anand et al., 2017). This difference might be related to the fact that the in the hospitalization stratum was reported an anomalous low rate of events, potentially reducing the benefit of spironolactone therapy in this subgroup. A probable explanation of this controversial data might be related to the geographical distribution of the patients enrolled: the majority of patients from Russia and Georgia were enrolled in the hospitalization stratum and thus were at lower risk, whereas those from the Americas were more balanced between the two strata and were at higher overall risk. In a *post hoc* analysis, spironolactone seemed to benefit patients in the Americas but not those in Russia or Georgia (Pitt et al., 2014).

Interestingly, Patel and colleagues using the data obtained from the OPTIMIZE-HF (Organized Program to Initiate Lifesaving Treatment in Hospitalized Patients with Heart Failure), a national registry of hospitalized HF patients evaluated the clinical effectiveness of aldosterone antagonists in older patients with HFpEF. The main outcomes of the study were a composite endpoint of all-cause mortality or HF hospitalization at 6 years of follow-up (primary outcome) and all-cause mortality, HF hospitalization, and all-cause hospitalization (secondary outcome). The authors were not able to find any association between aldosterone antagonists and all-cause mortality (HR: 1.03; *p* = 0.693) or HF hospitalization (HR: 0.88; *p* = 0.188) (Patel et al., 2012).

The first study to evaluate the effect if eplerenone in patient with HFpEf was the single-center RAAM-PEF study, in which Deswal and colleagues did not show any significant improvement in exercise capacity compared to placebo in the eplerenone group, but a significant reduction in markers of collagen turnover and improvement in diastolic function was detected in the latter (Deswal et al., 2011). More recently, Kampourides et al. evaluated the effect of eplerenone on the outcome of 303 patients (201 patients on eplerenone, 102 not on eplerenone treatment) with preserved systolic pump function (LVEF ≥40%) after ST-segment elevation acute myocardial infarction: the treatment with eplerenone was not able to demonstrate any impact on the occurrence of the primary endpoint of the study, i.e., death from cardiovascular causes, nonfatal reinfarction, hospitalization for unstable angina, or decompensation of heart failure. Remarkably, eplerenone was associated with a better prognosis only in patients with initially low MMP-9 levels, while no significant difference was detected in event-free survival rate in patients with high MMP-9 levels at baseline, suggesting that extracellular matrix turnover might play a key role in the natural history of HFpEF patients (Kampourides et al., 2012).

In the recent meta-analysis of 14 available RCTs, mineralocorticoid antagonist (MRA) reduced hospitalizations and improved diastolic function and quality of life, despite the lack of favorable effects on all-cause mortality (Chen et al., 2015). In the STRUCTURE trial, spironolactone significantly improved exercise capacity in parallel with the improvement in E/E′ ratio during exercise (Kosmala et al., 2016). Lastly, in another large meta-analysis of 15 RCTs, MRA administration was associated to a decreased risk of cardiovascular and all-cause mortality only in HFrEF patients, without any measured clinical benefit in HFpEF (Berbenetz and Mrkobrada, 2016).

In the Irbesartan in patients with heart failure and preserved ejection fraction (I-PRESERVE) trial, patients who were at least 60 years of age and had New York Heart Association class II, III, or IV heart failure and an ejection fraction of at least 45% were randomly assigned to receive 300 mg of irbesartan or placebo per day. The primary composite outcome was death from any cause or hospitalization for a cardiovascular cause (heart failure, myocardial infarction, unstable angina, arrhythmia, or stroke). During a mean follow-up of 49.5 months, the primary outcome occurred in 742 patients in the irbesartan group and 763 in the placebo group. Primary event rates in the irbesartan and placebo groups were 100.4 and 105.4 per 1,000 patient-years, respectively (hazard ratio, 0.95; 95% confidence interval [CI], 0.86–1.05; *p* = 0.35). Overall rates of death were 52.6 and 52.3 per 1,000 patient-years, respectively (hazard ratio, 1.00; 95% CI, 0.88–1.14; *p* = 0.98). When the authors evaluated the death modality, treatment with irbesartan did not impact on the overall mortality or the distribution of mode-specific mortality rates (Massie et al., 2008).

Consistently, in the CHARM-preserved trial, during a median follow up of 36.6 months, cardiovascular death did not differ between groups (170 vs. 170), but fewer patients in the candesartan group (*n* = 1,514, target dose 32 mg once daily) than in the placebo group were admitted to hospital for CHF once (230 vs. 279, *p* = 0.017) or multiple times. Composite outcomes that included non-fatal myocardial infarction and non-fatal stroke showed similar results to the primary composite (388 vs. 429; unadjusted 0.88 [0.77–1.01], *p* = 0.078; covariate adjusted 0.86 [0.75–0.99], *p* = 0.037). It is worthy of notice that the rates of study-drug discontinuation due to adverse events or laboratory abnormalities were significantly higher in the candesartan group (17.8 vs. 13.5%, *p* = 0.001), having the most common cause increase in creatinine level (4.8 vs. 2.4%, *p* = 0.0005), hypotension (2.4 vs. 1.1%, *p* = 0.009), and hyperkalemia (1.5 vs. 0.6%, *p* = 0.029). Another potential confounder is the fact that the statistical borderline significance in the reduction in HF hospitalizations was only reached after adjusting for covariates (*p* = 0.051 and *p* = 0.047, respectively) (Yusuf et al., 2003).

The study populations of I-PRESERVE and CHARM-preserved trial were highly representative of the HFpEF patients having a median EF of 59% and a mean EF of 54% respectively.

Therefore, even if there could be a pathophysiological rationale for RAAS targeting in HFpEF, clinical results are inconclusive, with more consistent advantages mainly seen on diastolic dysfunction, LV remodeling, and hospitalization events. Likely, spironolactone is beneficial only in selected subgroups of patients while ineffective in others; this theory was recently backed by another analysis of ALDO-DHF trial that linked specific collagen I phenotype, which exhibit extensive cross-links, with spironolactone resistance (Ravassa et al., 2018).

### Relaxin

In recent times, relaxin emerged as a promising therapeutic target in heart failure. This hormone acts on relieving vascular resistance mainly through RXFP1 receptor, localized on endothelium and vascular smooth muscle cells. It is demonstrated that its 48 h infusion in the animal model increases bradykinin-evoked NO-mediated relaxation and basal e-NO synthase protein expression (Leo et al., 2016). Moreover, prolonged administration of serelaxin, the recombinant form of relaxin, in animal model of heart failure improved vascular fibrosis and myocardial capillary density (McCarthy et al., 2017). These cardioprotective effects are mainly due to its ability to upregulate NOTCH signaling and inhibit TGF-B-SMAD 3 pathway (Sassoli et al., 2013). In another recent study in a diabetic animal model, *in vivo* serelaxin reversed endothelial dysfunction by enhancing NO-mediated relaxation thus improving ventricular hypertrophy and apoptosis (Ng et al., 2017). Even if promising, further studies in HFpEF are necessary and prospective validation of these findings in human subjects is required (Tschope et al., 2017; Dschietzig, 2019).

### A1-Agonists

The rationale behind the investigation of these drugs is based on the evidence that activation of the A1 adenosine receptor could improve mitochondrial function and contraction/relaxation coupling. The potential benefit of A1 agonist was partially contained in the observation of the detrimental effects associated with A1 receptor antagonism in the setting of HFpEF. Currently, one phase 2 trial (PANACHE) is evaluating the potential role of adenosine receptor stimulation in HFpEF (Voors et al., 2018). The experimental drug is neladenoson, a partial A1 receptor agonist. The proposed benefit hypothesized, targets cellular pathways typically dysregulated in myocardiocytes and endothelium – the improvement in mitochondrial function, which pairs with a reduced ROS production, the enhanced SERCA 2 activity preventing calcium overload, reduced lipotoxicity, decreased interstitial fibrosis, and hypertrophy and, finally, improved myocardial capillary density (Sabbah et al., 2013; Greene et al., 2016; Voors et al., 2018).

### Sacubitril/Valsartan

Even though the total amount of natriuretic peptides in these patients is lower than in HFrEF, this inhibitor of the neprilysin system is being investigated in HFpEF in the PARAGON HF trial, after data collected from the exploratory phase II PARAMOUNT study pointed toward a reduction in the total amount of natriuretic peptides with an improvement in NYHA class end renal function. Even if it is not known whether these effects are related to an improvement in microvascular function, the results of these studies are eagerly awaited (Shah et al., 2016).

### Sodium Glucose Co-transport-2 Inhibitors

A novel class of antidiabetic drugs has been recently introduced with putative salubrious effect in HF patients: the SGLT2 inhibitors (SGLT2i). Designed for patients with diabetes, reducing the kidney blood glucose resorption, rapidly SGLT2i gained attention among HF specialist since they were able to demonstrate a beneficial prognostic impact in patients affected by HF and diabetes, even independently from the glycemic control. Three SGLT2 inhibitors have been widely studied so far: canagliflozin, dapagliflozin, and empagliflozin. The EMPA-REG OUTCOME trial (EMPAgliflozin Cardiovascular OUTCOME Events in Type 2 Diabetes Mellitus Patients) included 7,020 patients with diabetes and established cardiovascular disease, showed a reduced risk of major CV events, CV mortality, all-cause mortality, and hospitalization for HF in 2TD patients, after a median observation time of 3.1 years when treated with empagliflozin. In particular, a 14% reduction in cardiovascular death, nonfatal myocardial infarction, nonfatal stroke and CV and all-cause mortality, despite a no significant difference about myocardial infarction or stroke. Patients treated with empagliflozin has a relevant reduction (−35%, *p* = 0.002) in hospitalization for HF compared with placebo group (Zinman et al., 2015). Similarly, canagliflozin decreased the 3 point-MACE by 14% (HR: 0.86, 95% CI: 0.75–0.97), which is a composite of CV death, non-fatal myocardial infarction, and non-fatal stroke in CANVAS (Neal et al., 2017).

CVD-REAL (Comparative Effectiveness of Cardiovascular Outcomes in New Users of SGLT-2 Inhibitors) is a large multinational study that analyzing data from six countries reported a lower risk of hospitalization and mortality for HF with several SGLT2i (Kosiborod et al., 2018). And even more recently, the DECLARE TIMI 58 study showed that the use of dapagliflozin resulted in a lower rate of hospitalization for heart failure when compared to placebo (hazard ratio, 0.73; 95% CI: 0.61–0.88); there was no difference between the groups in the rate of cardiovascular death (hazard ratio, 0.98; 95% CI: 0.82–1.17) (Wiviott et al., 2019). The current evidences do not support a widespread use of this class of drugs in patient with HF in the absence of diabetes. However, dissecting the mechanisms underlying these encouraging results might be crucial to design a trial program with the specific aim to fully legitimate their use in subset of patient affected by HF without diabetes (i.e., HFpEF and or HFmrEF).

### Ubiquinol and D-ribose

A novel target of research efforts has focused on this little electron transporter, moving from the hypothesis that supplementation of these molecules could yield to a more efficient ATP synthesis and cardiomyocyte performance, eventually reducing ROS production (Huynh et al., 2014). Indeed, in the frame of microvascular and, in general, microenvironmental dysfunction, coenzyme Q10 (CoQ10) depletion has been frequently identified, while its supplementation improved symptoms in HFrEF (Sharma et al., 2016). Moreover, Q10 decreased cardiac inflammation, fibrosis, and hypertrophy in animal models of diabetic cardiomyopathy (Huynh et al., 2010, 2013). A new trial on the topic is currently ongoing in this population (Pierce et al., 2018).

### Cardiospheres and Stem Cells

Cardiosphere-derived cells (CDCs) are a type of cardiac progenitor cells with anti-fibrotic, anti-inflammatory, and angiogenic properties (Smith et al., 2007; Malliaras et al., 2012; Tseliou et al., 2014; Aminzadeh et al., 2015). It is intriguing to notice that CDC proved effective in improving survival in the rat model of hypertension-induced HFpEF. These effects are seen irrespective of cardiac hypertrophy reversal or arterial blood pressure normalization. Instead, CDC increased the number of arterioles and capillaries, thus improving myocardial perfusion and coronary reserve (Gallet et al., 2015, 2016). This evidence further discloses the causal link between HFpEF, inflammation, and microvascular function. Another futuristic option in the setting of HFpEF is the potential use of stem cells – recently, in the animal model of diabetic cardiomyopathy, a disease phenotype which is very similar to HFpEF, bilayer of smooth muscle cells and endothelial progenitor cells, obtained from engineered bone marrow mesenchymal cells in rats, and implanted on the left ventricle, led to a preserved microvascular function through increased VEGF, decreased TGF-β and caspases expression, thus diminishing interstitial fibrosis while increasing microvascular density (Kawamura et al., 2017).

## Conclusions

The tantalizing research for a prognosis-improving therapy in HFpEF should not neglect the humbler attempt to enhance the quality of life, looking on the bright side of all those clinical trials failing in their primary endpoint but proving to relieve symptoms and reduce physical limitations. Albeit the novel paradigm, which puts the coronary microvascular endothelial inflammation on the culprit mechanistic pedestal and the common pathway in the complex HFpEF pathophysiology, the dismal outcomes revealed by RCTs testing one-therapy strategies prompt to perpetuate the research for pathophysiological subtypes.

The advance in mechanistic comprehension should aim to better understand the genetic and environmental etiological factors that might contribute to HFpEF onset and progression. This missing tile of the puzzle may facilitate the primary prevention of incidental HFpEF through accurate identification of vulnerable individuals and HFpEF-prone phenotypes. On the other hand, the discovery of more specific, reliable biomarkers may facilitate both early diagnosis and prognostic assessment.

## Author Contributions

DD, SM, JB, AR, NA, MG, RV, and FC conceived and wrote the manuscript. AL, GN, JB, RM, and SM reviewed the manuscript and made the final figure.

### Conflict of Interest

The authors declare that the research was conducted in the absence of any commercial or financial relationships that could be construed as a potential conflict of interest.
